# Hippo Signaling Pathway in Colorectal Cancer: Modulation by Various Signals and Therapeutic Potential

**DOI:** 10.1155/2024/5767535

**Published:** 2024-10-11

**Authors:** Somayeh Mohammadpour, Amir Torshizi Esfahani, SeyedKasra Sarpash, Fatemeh Vakili, Nikta Zafarjafarzadeh, Amirhesam Mashaollahi, Ali Pardakhtchi, Ehsan Nazemalhosseini-Mojarad

**Affiliations:** ^1^Basic and Molecular Epidemiology of Gastrointestinal Disorders Research Center, Research Institute for Gastroenterology and Liver Diseases, Shahid Beheshti University of Medical Sciences, Tehran, Iran; ^2^Department of Cellular and Molecular Biology, Faculty of Advanced Science and Technology, Medical Sciences, Islamic Azad University, Tehran, Iran; ^3^Gastroenterology and Liver Diseases Research Center, Research Institute for Gastroenterology and Liver Diseases, Shahid Beheshti University of Medical Sciences, Tehran, Iran

**Keywords:** colorectal neoplasms, hippo pathway, therapy, tumorigenesis, YAP/TAZ

## Abstract

Colorectal cancer (CRC) stands as a significant global health issue, marked by elevated occurrence and mortality statistics. Despite the availability of various treatments, including chemotherapy, radiotherapy, and targeted therapy, CRC cells often exhibit resistance to these interventions. As a result, it is imperative to identify the disease at an earlier stage and enhance the response to treatment by acquiring a deeper comprehension of the processes driving tumor formation, aggressiveness, metastasis, and resistance to therapy. The Hippo pathway plays a critical role in facilitating the initiation of tumorigenesis and frequently experiences disruption within CRC because of genetic mutations and modified expression in its fundamental constituents. Targeting upstream regulators or core Hippo pathway components may provide innovative therapeutic strategies for modulating Hippo signaling dysfunction in CRC. To advance novel therapeutic techniques for CRC, it is imperative to grasp the involvement of the Hippo pathway in CRC and its interaction with alternate signaling pathways, noncoding RNAs, gut microbiota, and the immune microenvironment. This review seeks to illuminate the function and control of the Hippo pathway in CRC, ultimately aiming to unearth innovative therapeutic methodologies for addressing this ailment.

## 1. Introduction

Colorectal cancer (CRC) ranks among the frequently detected malignancies across the globe, registering nearly 1.9 million fresh instances and 935,000 fatalities in the year 2020. The frequency and fatality proportions of CRC exhibit notable divergence among distinct global locales, showcasing escalated figures in more economically advanced nations [1]. During 2018, Asia recorded the greatest shares of occurrences (51.8%) and fatalities (52.4%) attributed to CRC across all genders and age groups, per 100,000 individuals [2].

The onset and progression of CRC entail a multifaceted journey influenced by genetic and environmental elements. The majority of CRC instances stem from precancerous growths referred to as adenomas, which have the potential to expand on the inner surface of the colon or rectum over the course of numerous years [3]. The progression from regular colonic epithelial cells to adenomas and eventually to carcinomas is instigated by the accrual of genetic mutations over time. Numerous genetic and environmental elements have been associated with the advancement of CRC. These factors encompass a familial precedent of CRC, an individual background of inflammatory bowel disease, obesity, smoking, alcohol usage, and a diet characterized by elevated red meat intake and inadequate fiber content [4]. The advancement and evolution of CRC encompass a multitude of signaling pathways, which encompass Wnt, PI3K/Akt, Hedgehog, ErbB, RHOA, Notch, BMP, AMP-activated protein kinase (AMPK), Hippo, NF-*κ*B, MAPK, and JNK. These pathways do not function in seclusion; rather, they engage in intricate interactions with each other. While AMPK, BMP, and Hippo pathways primarily exert anticancer effects in the context of CRC progression, other pathways serve as agents promoting carcinogenesis. These pathways interact not solely within the cellular cytoplasm but also extend to the nucleus, thus governing the transcription of downstream genes that contribute to cancerous cell proliferation, inhibition of apoptosis, mobility, invasion, and cellular dedifferentiation [5].

In 1995, Pan [6] and his team introduced the Hippo signaling pathway via a genetic screening technique. Their aim was to identify mutations within *Drosophila melanogaster*, the widely known fruit fly species, that led to unusual tissue proliferation. The scientists chose the name “Hippo” pathway due to the resemblance between the overgrown imaginal discs in the mutated flies and the head of a hippopotamus [6]. Later investigations showcased that the Hippo pathway is conserved across evolutionary lines, extending to mammals such as humans. It assumes a vital role in governing cell growth, tissue stability, control of organ size, proliferation, differentiation, and apoptosis in diverse tissues and organs [7]. The malfunctioning of the Hippo pathway has been implicated in the genesis of a range of disorders, cancer being one of them [6]. The observation that anomalies in the Hippo gene could lead to excessive tissue overgrowth in *D. melanogaster* prompted the speculation that the disruption of the Hippo pathway, a factor linked to various diseases, including cancer [6, 8, 9], might have an intimate connection with cancer development [10–12].

In the context of human cancers, the Hippo pathway holds sway over the initiation and advancement of tumors, the spread of tumors to other sites, and the resistance of tumors to therapeutic drugs, covering these three principal domains of influence. This review will delve into the fundamental constituents, signals originating upstream, and essential physiological processes overseen by the Hippo pathway. Moreover, it will scrutinize research endeavors exploring the repercussions of an aberrant Hippo pathway and evaluate prospective therapeutic approaches aimed at targeting Hippo pathway constituents within CRC.

## 2. The Core of Hippo Signaling Pathway

In mammals, the Hippo pathway is composed of several vital components, which encompass MST1/2 (sterile 20-like 1/2), SAV1 (Salvador homolog 1), LATS1/2 (large tumor suppressor kinase 1/2), MOB1A/B (MOB kinase activator 1A/B), and YAP/TAZ (yes-associated protein/transcriptional co-activator with PDZ binding motif) [13].

**Figure 1:**
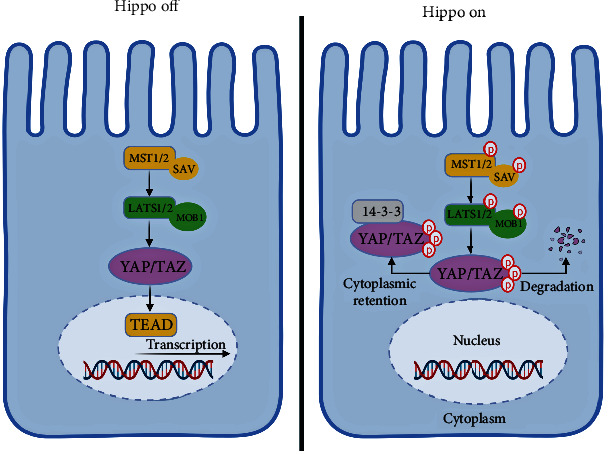
The Hippo signaling pathway. The core mechanism of the Hippo signaling pathway involves the intricate orchestration of phosphorylation driven by several upstream signals. Upon pathway activation, MST1/2 kinases and SAV1 collaborate to form a complex that executes phosphorylation and activates LATS1/2. Subsequently, the downstream actors of this pathway, namely YAP/TAZ proteins, undergo phosphorylation by LATS1/2 kinases. This triggers the recruitment of 14-3-3 proteins, facilitating their sequestration in the cytoplasm or triggering their degradation. When the Hippo signaling pathway deactivates, YAP/TAZ undergo dephosphorylation, facilitating their movement into the nucleus, where they form a partnership with the TEADs transcription factors. Together, this assemblage regulates the genes essential for endothelial cell proliferation, migration, and survival. The constituents include MST1/2 (mammalian Ste20-like kinase), SAV1 (scaffold protein salvador), LATS1/2 (large tumor suppressor kinase), TAZ (transcriptional coactivator with PDZ-binding motif), YAP (yes-associated protein), and TEAD (TEA domain family member).

Signals like cell density and mechanical tension initiate the Hippo pathway, which triggers the activation of upstream kinases MST1/2 and SAV1. These kinases phosphorylate and activate downstream kinases LATS1/2 (as depicted in Figure 1). Scaffolding proteins Sav/Rassf/Hpo and MOB1A/B also play a crucial role in activating LATS1/2. The family of MAP4K proteins can also activate LATS1/2 or employ other kinases. LATS1/2 phosphorylates the transcriptional co-activators YAP and TAZ, limiting their nuclear localization and function [14].

YAP/TAZ's phosphorylation leads to binding with 14-3-3 proteins, impeding their passage into the nucleus. However, when the Hippo pathway encounters inhibition, YAP/TAZ can migrate to the nucleus and activate genetic patterns that foster cell growth and viability. WW domains in YAP/TAZ connect with transcription factors like TEAD1-4, which play a pivotal role in YAP/TAZ's oncogenic role [15]. YAP1 activation can induce growth-promoting traits, ultimately driving tumor progression [16].

The STRIPAK complex is crucial in controlling the Hippo pathway by affecting LATS1/2 and YAP/TAZ. It consists of STRN, PP2A, and MST3. STRN and PP2A interact with LATS1/2, triggering their dephosphorylation, and MST3 can connect with SAV1 and inhibits MST1/2′s activities, thereby inactivating the Hippo pathway [17].

MOB1A/B plays a dual role in the Hippo pathway activation. It facilitates the activation of downstream kinases LATS1/2, leading to the phosphorylation and inhibition of YAP and TAZ's nuclear localization and transcriptional functions. It also interacts with MST1/2, the upstream kinase responsible for activating LATS1/2. Thus, MOB1A/B acts as a co-factor for both upstream and downstream kinases within the Hippo pathway, playing a pivotal role in its activation [18].

Another important regulator of the Hippo pathway is VGLL3, which acts as a transcriptional co-activator of TEAD proteins, promoting the transcription of genes that inhibit cell growth and proliferation [19]. VGLL3 and TEAD proteins interact, maintaining tissue homeostasis and preventing tumorigenesis, as they compete with YAP/TAZ for binding and upregulating expression upon Hippo pathway activation [20].

The Hippo pathway is a complex signaling mechanism in mammalian cells that regulates transcriptional coactivators like YAP/TAZ, promoting cell growth and survival. Its activation and deactivation are regulated through various processes, including phosphatase activity, cofactor binding, autophosphorylation, and negative feedback loops. Increased nuclear YAP1 levels have been found in solid tumors, and various Hippo pathway constituents have been altered in human cancer at the DNA level, including YAP1 and TAZ gene amplification, deletions, or truncating mutations in NF2, LATS1, and LATS2 gene [21, 22]. Research on YAP1 and TAZ has shown that these transcriptional cofactors have a multifaceted role in tumor growth. They can control the tumor microenvironment (TME), impact antitumor immunity, induce drug resistance, and function as inherent oncogenic drivers within tumors. They also have the ability to induce resistance to various drugs, both cytotoxic and targeted agents. This suggests that these transcriptional cofactors have a significant impact on tumor growth and its regulation [23–25]. In a recent cellular screening, the activation of YAP1 was discovered as a novel mechanism associated with resistance to a triple combination of EGFR, BRAF, and MEK inhibitors (cetuximab, dabrafenib, and trametinib) in cell lines characterized by BRAF mutant CRC [26]. Furthermore, a comprehensive genome-scale cDNA screening detected the activation of YAP1 as a determinant for cell survival following KRAS knockdown in CRC cell lines carrying the KRAS G13D mutation [27]. Nevertheless, a separate study has presented evidence suggesting that YAP can impede Wnt signaling during the phases of intestinal regeneration and the progression of tumor development [28]. According to the results of an independent study, a robust correlation was observed between the elevated expression of TAZ in CRC tissues and the progression of cancer, as well as an adverse prognosis [29]. There is ample evidence to support a positive correlation between YAP expression and poor overall survival (OS) in CRC [30, 31]. In contrast, the results of a study indicate that loss of YAP expression correlates with a poor prognosis and may represent a subgroup with more aggressive biological features in CRC [32]. Therefore, it is not feasible to categorize YAP as solely a tumor promoter or a tumor suppressor, as its role in tumor development and progression is complex and context-dependent [33].

## 3. Upstream Regulators of Hippo Signaling Pathway in CRC

**Figure 2:**
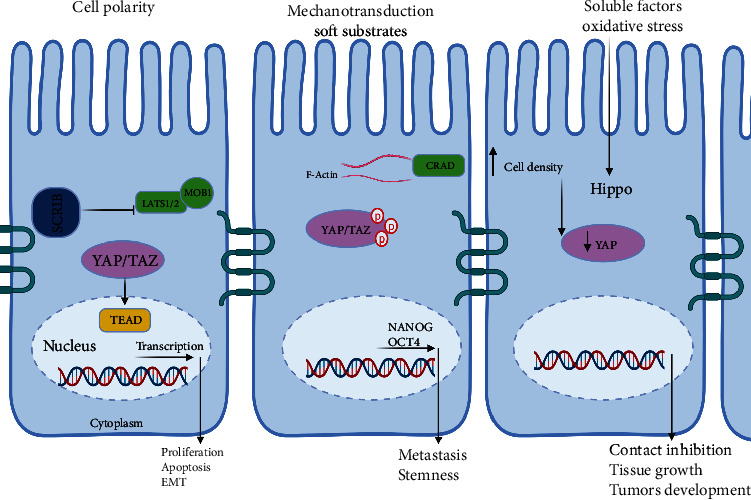
Upstream regulators of Hippo signaling pathway in CRC. The Hippo can be activated by diverse stimuli including cell polarity, mechanotransduction, cell density, soluble factors, and oxidative stress. Upstream regulators of Hippo signaling pathway in CRC. The Hippo can be activated by diverse stimuli including cell polarity, mechanotransduction, cell density, soluble factors, and oxidative stress. Cell polarity proteins like AMOT and scribble complexes regulate Hippo pathway components such as LATS1/2 and YAP/TAZ. Mechanical cues from the tumor microenvironment are transduced to the Hippo pathway via actin cytoskeleton rearrangements and activation of transcription factors. High cell density activates the tumor suppressor NF2 to inhibit YAP. Soluble factors like EGF, TGF-*β*, and IGF-1 modulate Hippo pathway kinases and transcriptional coactivators. Oxidative stress can both activate and inhibit the Hippo pathway through redox signaling. Dysregulation of these upstream signals leads to aberrant Hippo pathway activity and CRC progression.

The Hippo signaling pathway distinguishes itself from typical ligand–receptor transduction pathways by its capacity to integrate diverse upstream cues. These encompass factors like cell polarity, mechanotransduction, soluble substances, cell–cell adhesion, mechanical tension stemming from the actin cytoskeleton, and the force transmitted from the extracellular matrix (ECM), as illustrated in Figure 2.

### 3.1. Cell Polarity

Cell polarity, crucial for maintaining tissue architecture and suppressing tumorigenesis, plays a pivotal role in Hippo pathway regulation [34]. Within this pathway, various proteins at cell junctions, including angiomotin (AMOT), *α*-catenin, and zonula occludens (ZO) proteins, are instrumental in modulating signaling cascades [35]. Moreover, planar cell polarity (PCP), mediated by the interaction between protocadherins Fat (Ft) and Dachsous (Ds), contributes significantly to Hippo pathway regulation, with the steepness of Ds–Fj gradients dictating pathway activity [36, 37].

In CRC, disruptions in cell polarity are closely associated with metastatic progression [38]. Specifically, the dysregulation of scribble complexes, crucial regulators of cell polarity, is observed in CRC. Elevated expression of scribble (SCRIB) is frequently encountered in CRC patients and correlates with aggressive malignancy traits, such as heightened proliferation, apoptosis resistance, and epithelial-mesenchymal transition (EMT) [39]. Mechanistically, increased SCRIB activity suppresses the Hippo pathway by downregulating LATS1/2 and MOB1A/B, consequently impeding YAP phosphorylation. As a result, unphosphorylated YAP translocates into the nucleus, where it activates transcription of target genes including TEAD1–4, thereby promoting CRC progression [40].

### 3.2. Mechanotransduction

Mechanical cues, including matrix stiffness, fluid flow, and cellular forces, influence tumor growth and metastasis, alongside biochemical factors like hypoxia and angiogenesis [41, 42]. Resistance to anti-angiogenic therapy in colorectal liver metastasis may increase due to heightened ECM stiffness [39]. Targeting mechanobiological pathways via mechanotherapy holds promise for tackling CRC [41, 43].

The Hippo pathway, a mechanotransduction hub, integrates mechanical signals to regulate tissue homeostasis and organ size. Dysregulation of this pathway contributes to cancer development by promoting uncontrolled cell proliferation and inhibiting apoptosis [44]. Mechanical cues, like cell–cell contact and cell–matrix interactions, modulate core Hippo pathway components [45].

Mechanical force can activate the Hippo pathway via GPCR-mediated RhoA-ROCK signaling or through cytoskeleton regulation [46]. Newer studies have revealed that the Hippo pathway undergoes regulation through the combined interactions of F-actin architecture, cellular morphology, and the external environment [47]. Actin polymerization positively regulates Yki/YAP activity, with upstream regulators like Merlin and expanded coordinating cell proliferation [48].

Cell morphology and mechanical stress influence the Hippo pathway, with Rho activity blockade eliminating their impact [49]. Additionally, AMOT and the actin complex receive regulation from Mer, linking the plasma membrane and actin filaments [50], while actomyosin filaments, via the LINC complex, directly connect to the nuclear envelope, governing Yki/YAP activity [51].

In CRC, mechanotransduction plays a critical role in tumor development and progression, linking the tension-dependent actomyosin skeletal mechanotransduction to CRC cell deformability and metastasis. This connection may involve the capping protein inhibiting regulator of actin dynamics (CRAD), whose reduced levels on soft substrates retain YAP in the cytoplasm, restoring repression on stemness markers like NANOG and OCT4. CRAD's involvement suggests its contribution to CRC stemness and metastasis, underscoring the significance of the mechanotransductive axis CRAD-F-actin-YAP [52]. Additionally, matrix stiffness influences CRC cell stemness via the YAP and integrin*β*1/FAK pathway, which is dephosphorylated in response to matrix stiffness [53].

In summary, the complex interplay between mechanical cues, the Hippo pathway, and CRC underscores the need for further research to develop effective mechanotherapy options and gains insights into cancer biology.

### 3.3. Cell Density

The Hippo pathway regulates cell density, crucial for tissue balance. In health, it curbs proliferation and triggers apoptosis with rising density. Disrupted in cancer, it drives uncontrolled growth, often via mutations or regulator expression changes. Cell–cell adhesion and mechanics also affect its function [8].

When cells reach high density, large tumor suppressor kinase (LATS) activates, phosphorylating and inhibiting YAP, a key pathway effector [54]. Several proteins, including crumbs complex members and KIRREL1, act as sensors, transmitting density cues to Hippo [55–59]. For instance, KIRREL1 aids SAV1 recruitment to cell–cell contact sites, regulating Hippo feedback [56]. Additionally, TEAD, a YAP target, is palmitoylated in a density-dependent manner [60].

New research has emphasized the significance of cell density in controlling the Hippo signaling pathway in CRC. In particular, the expression of YAP reduced as cell density increased in human CRC samples, which was reliant on the expression of the tumor-suppressor NF2. NF2 functions as a sensor in the cell-to-cell contact inhibition of the Hippo signaling pathway, and its levels increase as cancer cell density rises. Therefore, the regulation of YAP by cell density implies a crucial role of the Hippo pathway in contact inhibition, tissue growth, and the development of tumors in CRC [61].

To conclude, the Hippo pathway assumes a vital role in processes like contact inhibition, tissue growth, and the development of tumors, particularly in the context of CRC.

### 3.4. Soluble Factors

Soluble factors like growth factors, cytokines, and hormones play crucial roles in cellular behavior regulation, impacting cell proliferation, differentiation, migration, and survival. Dysregulated signaling of these factors is common in cancer, with the Hippo pathway emerging as a key player in organ size control, tissue equilibrium, and tumorigenesis [13]. For instance, the tumor suppressor Merlin activates the Hippo pathway but is negatively regulated by epidermal growth factor (EGF), leading to YAP/TAZ activation and promoting cancer growth and invasion [62]. Other soluble factors like transforming growth factor beta (TGF-*β*) and insulin-like growth factor 1 (IGF-1) also modulate the Hippo pathway, impacting cancer development and progression [63, 64]. TGF-*β*, for example, activates the Hippo pathway by stimulating LATS kinases, resulting in YAP/TAZ inactivation and suppressing cell proliferation and migration [65].

There are several soluble factors that have been implicated in CRC, including IL-4, MIP-1*β*, FasL and TGF-*β*1, IL-8, and VEGF [66]. The Hippo pathway in CRC can be regulated by the soluble factors through the regulation of downstream effector proteins. As a result, the modulation of the Hippo pathway by these factors is vital in the development and advancement of CRC. Therefore, targeting the upstream regulators or the Hippo pathway could be a promising therapeutic approach for treating CRC.

### 3.5. Oxidative Stress

The Hippo pathway, critical for cellular growth regulation, is implicated in various cancers due to its dysfunction. It responds to stressors like reactive oxygen species (ROS), mechanical stress, and DNA damage, orchestrating processes such as apoptosis, pyroptosis, and metastasis [67].

One ROS-induced Hippo pathway activation mechanism is the upregulation of the redox protein thioredoxin (Trx), which can mediate YAP upregulation and thereby control redox status [68]. Additionally, ROS can phosphorylate eIF2, activating the ATF-NEED4.2 chain and inhibiting the Hippo pathway [69]. Conversely, YAP and FOXO1 establish functional complexes on antioxidant gene promoters, regulating redox signaling [70]. YAP also has the capability to associate with FoxM1, facilitating the enhancement of Nuclear factor erythroid 2-related factor 2 (Nrf2) expression, a specific type of antioxidant factor [71].

Crosstalk between Hippo and redox pathways involves proteinase-activated receptor 2 (PAR2) signaling, miR-25-mediated LATS suppression, and YAP's modulation of Bcl-2 under oxidative stress [72–74]. Furthermore, Nrf2 regulates antioxidant gene expression, influencing Hippo pathway activity [75, 76]. Oxidative stress contributes to CRC development by damaging DNA and promoting adenoma–carcinoma progression [77]. Gut microbiota members can also increase CRC risk by generating oxidants [78]. Dysregulation of Hippo-redox interplay affects apoptosis, cell survival, and CRC progression, highlighting its therapeutic potential and warranting further research. Drug resistance in CRC due to redox adaptation poses a significant challenge, mediated by increased antioxidant expression, DNA repair, and inhibition of apoptosis [79–81]. Overexpression of FGFR4, SOD variants, and DUOX can induce chemoresistance [82].

Understanding ROS's role in CRC drug resistance is crucial for developing effective strategies to overcome this challenge.

## 4. Crosstalk Between Hippo Signaling Pathway and Other Cellular Signaling Pathways in CRC

**Figure 3:**
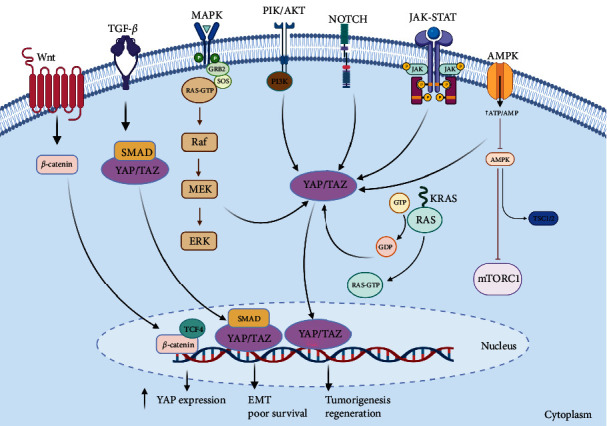
Schematic model of the Hippo pathway and crosstalk with other signaling pathways in CRC. The core Hippo pathway involves a kinase cascade leading to YAP/TAZ regulation. Hippo pathway interacts bidirectionally with Wnt, TGF-*β*, PI3K/AKT, Notch, and other oncogenic signaling pathways. These interactions influence CRC cell proliferation, survival, stemness, and metastasis. Targeting these intersections may provide therapeutic approaches.

Aberrations in the Hippo signaling pathway have been implicated in the initiation and advancement of diverse cancer types. The interplay between the Hippo signaling pathway and other cellular signaling pathways is intricate and contingent upon the specific context. Below, you will find an in-depth examination of the interactions between the Hippo signaling pathway and other cellular signaling pathways within the realm of CRC (Figure 3).

### 4.1. Wnt Signaling Pathway

The crosstalk between the Wnt signaling pathway and the Hippo signaling pathway has been extensively studied in cancer, and it is now recognized as a key factor in cancer development and progression. Here, we will provide a more detailed analysis of this crosstalk in the context of cancer, including CRC [83].

The activation of the Wnt signaling pathway is commonly observed in various cancers, including CRC, hepatocellular carcinoma, breast cancer, and pancreatic cancer [84, 85], often due to mutations in APC and *β*-catenin genes, leading to *β*-catenin stabilization and subsequent Wnt target gene activation [86]. Recent studies have revealed that the Hippo pathway can modulate the Wnt pathway by regulating *β*-catenin stability and activity [87–89]. AP/TAZ interact with *β*-catenin, promoting its nuclear localization and activating Wnt target genes [90], while also regulating expression of Wnt pathway components, including receptors and the antagonist Dkk1 [91]. This crosstalk is particularly relevant in cancer stem cells (CSCs), where both pathways regulate CSC maintenance and tumor initiation. YAP/TAZ induce Wnt ligand and receptor expression, promoting Wnt signaling in CSCs [92].

Both microsatellite stable (MSS) CRCs without hypermutation and microsatellite instability (MSI) CRCs with hypermutation display aberrant activation of the Wnt signaling pathway [93]. Elevated expression of YAP and *β*-catenin is observed in primary colon cancer tumors. The binding of *β*-catenin/TCF4 complexes to a DNA enhancer element within the first intron of the YAP gene induces YAP expression in CRC cells. When *β*-catenin expression in CRC cells is reduced using shRNAs, there is a corresponding decrease in YAP mRNA and protein levels. The localization pattern of YAP in the cytoplasm and nuclei of various human colon cancer cell lines is insensitive to plating density. These findings suggest that YAP functions as an oncogene in human CRC cells, with its expression being driven by aberrant Wnt/*β*-catenin signaling [94].

The complex crosstalk between Wnt and Hippo pathways in cancer presents a promising therapeutic target for CRC. Several Wnt and Hippo signaling inhibitors are currently under development and in clinical trials, and their efficacy and safety are being evaluated in cancer patients [95, 96]. The success of these inhibitors will depend on a better understanding of the crosstalk between the Wnt and Hippo pathways and their roles in cancer development and progression.

### 4.2. TGF-*β* Signaling Pathway

The Hippo signaling pathway and the TGF-*β* signaling pathway are both involved in regulating cell proliferation, differentiation, and apoptosis, and their dysregulation is implicated in cancer development and progression. The two pathways crosstalk in several ways, including through the regulation of transcription factors and downstream effectors.

Indeed, both MSS CRCs lacking hypermutation and MSI CRCs with hypermutation exhibit irregular activation of the Wnt signaling pathway [97]. The tight regulation of the nuclear-cytoplasmic shuttling of SMADs involves their association with YAP. If YAP/TAZ are absent, the accumulation of SMADs in the nucleus is hindered, resulting in the inhibition of TGF-*β* mediated transcription [98]. In a context-dependent manner, the interactions between Smad2/3-mediated transcription and TAZ/YAP appear to be both positive and negative regulators. Smad2/3, TEAD4, and TAZ/YAP can form a complex with OCT4, leading to the transcription of pluripotency genes while suppressing mesendodermal differentiation. Disruption of this complex through the co-regulation of the Smad2/3-Smad4 complex and FOXH1 promotes the induction of mesendodermal genes [99].

Numerous research studies have indicated intercommunication between the Hippo and TGF-*β* signaling pathways in CRC. One such instance is the observation that TAZ mRNA is significantly amplified in consensus molecular subtype 4 (CMS4) tumors, which are associated with the expression of EMT, TGF-*β*, and poorer survival outcomes. Conversely, CMS2 and CMS3 tumors have lower TAZ expression levels [100]. Moreover, findings from a separate study demonstrated a connection between TAZ expression and the TGF-*β* signaling pathway, which is known to modulate the production of ECM, tissue fibrosis, and inflammation [29].

In summary, crosstalk between the Hippo and TGF-*β* signaling pathways is important for CRC development and progression. These pathways interact to regulate various cellular processes, including cell proliferation, migration, and invasion. Understanding the mechanisms underlying this crosstalk may lead to developing novel therapeutic strategies for CRC.

### 4.3. Notch Signaling Pathway

The interplay between the Hippo and Notch signaling pathways has been demonstrated to be intricate and multifaceted across different cancer types. This interaction between the pathways assumes a noteworthy role in governing critical cellular processes like cell proliferation, differentiation, and stemness.

One of the key modes of interplay between the Hippo and Notch pathways revolves around the modulation of the downstream mediator YAP/TAZ. Evidence highlights that YAP/TAZ holds the capacity to transcriptionally govern Notch receptors and ligands, thereby facilitating cis-inhibition within neighboring cells and preserving an undifferentiated status. When YAP/TAZ is absent, the suppression on Notch signaling is lifted, ultimately prompting cellular differentiation [101]. Research findings have also indicated the existence of a positive feedback loop connecting YAP/TAZ and Notch signaling. In this loop, YAP/TAZ facilitates the transduction of Notch signaling by inducing the expression of Jag1. Conversely, Notch signaling counteracts the degradation process mediated by *β*-Trcp, thereby stabilizing the TAZ protein [102].

The proper functioning of Notch1 signaling is essential for maintaining intestinal homeostasis. However, when Notch1 signaling is abnormally activated, either through ligand-dependent or independent mechanisms, it disturbs the delicate balance of regulatory pathways mediated by Notch1, ultimately resulting in the promotion and proliferation of CRC [103]. A study uncovered a vital function of Notch, YAP, and the histone methyltransferase Mll1 in both CRC tumorigenesis and regeneration. The results indicate that the constant activation of Notch in cells that lack p53 leads to the promotion of Yap and Mll1, causing cells to shift to a regenerative state, driving niche-factor-independent growth, and making cells susceptible to tumorigenesis if the state persists [104].

Overall, the interplay between the Hippo and Notch pathways is intricate and influenced by the context, and additional investigation is necessary to grasp their interactions in the development and progression of CRC.

### 4.4. PI3K/AKT Signaling Pathway

The Hippo signaling pathway has been shown to interact with the PI3K/AKT signaling pathway in various types of cancer. PIK positively regulates YAP/TAZ to inhibit mammary cell death, promotes transformation *in vitro*, and enhances YAP/TAZ activity in mammary tumorigenesis *in vitro*. The regulation of YAP/TAZ by PIK3 occurs through multiple signaling pathways, including LATS-dependent and LATS-independent pathways. These results suggest a crosstalk between PI3K and YAP/TAZ, promoting breast cancer cell transformation [105].

The Hippo pathway controls YAP/TAZ signaling in normal tissues, and its core components include MST1/2 and LATS1/2 kinases that coordinate organ growth and homeostasis, and MST1/2 or MAP4K4 activates LATS kinases through phosphorylation [106]. The active control of the Hippo pathway leads to YAP/TAZ inactivation by LATS kinases, which phosphorylate them, retain them in the cytoplasm, and degrade them [107]. However, the lack of inhibition by the Hippo pathway promotes the translocation of YAP/TAZ into the nucleus, where they bind to TEAD proteins to form YAP/TAZ-TEAD complexes. This mechanism promotes the loss of contact inhibition of cell growth in cancer cells in a PI3K-dependent way [108]. The activation of PI3K and phosphoinositide-dependent kinase-1 (PDK1) following the stimulation of the EGF receptor triggers a sequence of events. This includes the disassembly of the Hippo core complex, the subsequent inactivation of LATS, the dephosphorylation of YAP, and the consequent accumulation of YAP in the nucleus, leading to its transcriptional activation [108]. In addition, AKT can increase the activity of YAP proteins by phosphorylating MST1/2, inhibiting their dimerization and activation [109]. YAP/TAZ-TEAD complexes cooperate with other transcription factors to regulate gene transcription, and they can also bind to other transcriptional factors, such as *β*-catenin, to upregulate the expression of genes promoting EMT and cell survival [110].

PI3K/AKT pathway is a significant signaling cascade in colon cancers. Its activation has been documented in 60%–70% of CRCs, and various studies have proposed inhibitors of pathway components as potential therapeutic agents [111]. The crosstalk between the Hippo and PI3K/AKT pathways in CRC highlights the complexity of signaling pathways involved in cancer progression and suggests potential targets for therapeutic intervention.

### 4.5. MAPK Signaling Pathway

The Hippo signaling pathway and the mitogen-activated protein kinase (MAPK) signaling pathway are two important intracellular signaling pathways that play a critical role in regulating various cellular processes, including cell proliferation, differentiation, and survival [112]. In recent years, it has become increasingly clear that these two pathways can crosstalk with each other, and dysregulation of this crosstalk is frequently observed in cancer.

One way the Hippo and MAPK pathways can crosstalk is by regulating the downstream effector YAP/TAZ. MAPK signaling can activate YAP/TAZ by inhibiting the upstream Hippo kinases MST/LATS, leading to the accumulation of YAP/TAZ in the nucleus and promoting cell proliferation and survival. Conversely, YAP/TAZ can also activate the MAPK pathway by inducing the expression of growth factors and their receptors [113]. Another crosstalk mechanism between these two pathways is the regulation of the transcription factors AP-1 and ETS [113]. MAPK signaling can activate AP-1 and ETS [114], which can in turn activate the expression of YAP/TAZ target genes. Additionally, YAP/TAZ can also interact with AP-1 and ETS to regulate gene expression [113].

In CRC, MAPK pathways are frequently detected to be overexpressed and activated, significantly contributing to cancer progression. Therefore, MAPK pathways could serve as a promising molecular target for treating this disorder [115].

### 4.6. JAK/STAT Signaling

The Hippo signaling pathway and JAK/STAT signaling pathway are both essential for the regulation of cellular processes such as proliferation, differentiation, and apoptosis. The Hippo pathway is known to regulate organ size and tissue homeostasis, while JAK/STAT signaling is involved in immune responses and hematopoiesis [116].

The expression of TAZ is stimulated by type I interferon, and it exerts a negative regulatory effect on JAK-STAT signaling by inhibiting the nuclear localization of STAT1/2 and the expression of IFN-stimulated genes [117].

The exploration of the JAK/STAT pathway as a therapeutic target has gained prominence for the treatment of CRC. Various inhibitors of JAK/STAT signaling have been formulated and are currently undergoing assessment in clinical trials to ascertain their effectiveness in managing CRC. These inhibitors have exhibited encouraging outcomes in preclinical investigations, manifesting their ability to hinder tumor growth, curtail cell proliferation, and stimulate apoptosis within CRC cells [118, 119].

Further research is needed to understand the underlying mechanisms of this crosstalk and to identify potential therapeutic targets for cancer treatment.

### 4.7. AMPK Pathway

The AMPK and Hippo signaling pathways play important roles in regulating cellular metabolism, proliferation, and survival [120]. Recent studies suggest that crosstalk between these two signaling pathways may be involved in the development of CRC. AMPK is a cellular energy sensor that is activated in response to low ATP levels or increased AMP: ATP ratios. It regulates energy metabolism by promoting catabolic pathways and inhibiting anabolic pathways [121]. Conversely, the Hippo signaling pathway governs functions such as organ dimensions, tissue equilibrium, and cellular proliferation through the modulation of the activity of transcriptional co-activators YAP/TAZ [122].

Several studies have shown that the AMPK and Hippo signaling pathways interact with each other at multiple levels in CRC. AMPK can inhibit the Hippo pathway by phosphorylating YAP at serine 94, which promotes its cytoplasmic retention and degradation [123]. Alternatively, the Hippo–YAP signaling pathway can exert control over AMPK energy stress via the AMPK-mediated modulation of AMOT like-1 protein (AMOTL1). Energy stress, brought about by factors like nutrient deficiency or pharmacological AMPK activation, prompts the relocation of AMOTL1 to the cell membrane, which subsequently curtails YAP's activity. These discoveries unveil a fresh mechanism for the regulation of Hippo–YAP signaling in response to energy stress, thereby identifying potential targets for therapeutic intervention in disorders associated with Hippo–YAP irregularities [124]. YAP enhances the expression of Glut3, a glucose transporter, which increases the uptake of glucose by the cancer cells. This, in turn, activates the AMPK signaling pathway, which promotes cell proliferation and migration. The study suggests targeting YAP or its downstream signaling pathways could be a potential therapeutic strategy for treating CRC [125].

Moreover, dysregulation of either pathway can lead to the development of CRC. For example, the loss of LKB1, a negative regulator of YAP/TAZ, has been shown to promote CRC growth by activating the Hippo pathway [125]. Similarly, activation of the AMPK pathway has been shown to inhibit CRC growth by promoting cell cycle arrest and apoptosis [126].

To summarize, the interplay between the AMPK and Hippo signaling pathways holds significant importance in the progression of CRC. There is a need for more comprehensive investigations to uncover the precise molecular mechanisms that underlie this interaction, with the aim of formulating targeted therapies for CRC that leverage this interplay.

### 4.8. KRAS Signaling

KRAS, a constituent of the RAS family of small GTPases, assumes a pivotal role in cellular signaling pathways governing essential functions like cell growth, differentiation, and survival. Within the spectrum of genetic changes in cancer, mutations in KRAS hold prominence, particularly in pancreatic, colorectal, and lung cancers. These mutations induce the constant activation of the KRAS protein, culminating in unregulated cell proliferation [127].

The transcriptional coactivator YAP1 emerges as a pivotal factor in the viability and transformation of KRAS-dependent cells. Its heightened signaling contributes to the development of resistance to KRAS inhibition in lung cancer. YAP1 and KRAS collaborate to activate the transcription factor FOS, which governs the process of EMT. This underscores the significance of YAP1-mediated transcriptional control within the context of oncogenic RAS signaling [27].

An additional mode through which KRAS suppresses the Hippo pathway involves the manipulation of the actin cytoskeleton. The actin cytoskeleton is an intricate arrangement of protein filaments that offers structural reinforcement to cells and holds pivotal roles in processes like cell migration, division, and signaling. Activation of KRAS triggers a reconfiguration of the actin cytoskeleton [128], subsequently impeding the Hippo pathway by disturbing the localization of its key components to the cell membrane [129].

The crosstalk between KRAS and Hippo signaling pathways in cancer is complex and involves multiple mechanisms. Dysregulation of these pathways can lead to uncontrolled cell growth and proliferation, and targeting these pathways may provide new opportunities for cancer therapy.

Recent studies have shown crosstalk between the KRAS and Hippo signaling pathways in CRC. In particular, the activation of certain amino acid transporters can be induced by oncogenic KRAS mutations through the YAP1 pathway of hippo signaling, resulting in mTOR activation and the proliferation of CRC cells [130]. RASAL2, a protein involved in cell signaling pathways, promotes the progression of CRC by activating the LATS2/YAP1 axis of the Hippo signaling pathway [131]. The RAF–MEK–ERK signaling pathway is activated by KRAS mutations and has been shown to crosstalk with the Hippo pathway in CRC. Activated RAF–MEK–ERK signaling can phosphorylate and inactivate LATS1/2, which are key components of the Hippo pathway. This leads to the activation of YAP/TAZ and increased cell proliferation and survival [132].

In summary, crosstalk between the KRAS and Hippo signaling pathways plays an important role in the development and progression of CRC. Activation of YAP/TAZ and inactivation of LATS1/2 by the KRAS pathway leads to increased cell proliferation and survival. Understanding the crosstalk mechanisms between these two pathways may lead to developing novel therapeutic targets for treating CRC.

## 5. Crosstalk Between Hippo Signaling Pathway and Noncoding RNAs (ncRNAs) in CRC

ncRNAs encompass RNA molecules that lack the capacity to encode proteins but exert critical regulatory functions within cells. The dichotomous role of microRNAs (miRNAs), whether as tumor suppressors or oncogenes, hinges on their interactions with target mRNAs. These interactions, in turn, exert a consequential impact on the cellular phenotype [133]. CRC features the involvement of various ncRNAs in its progression and development. Among these, miRNAs, typically measuring 21–23 base pairs in length, have been extensively examined in CRC. Another noteworthy category is long noncoding RNAs (lncRNAs), which exceed 200 nucleotides in length and have been implicated in CRC. Beyond miRNAs and lncRNAs, other ncRNA varieties like circular RNAs (circRNAs) and small nucleolar RNAs (snoRNAs) have also emerged as players in CRC. CircRNAs, characterized by covalently closed loops, exert regulatory control over gene expression by acting as miRNA sponges [134].

**Figure 4:**
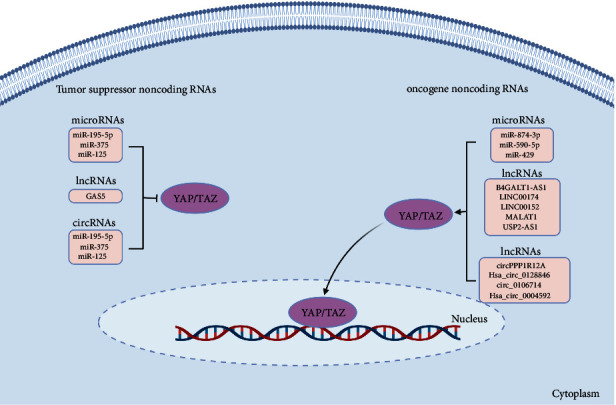
Schematic model of the Hippo pathway and crosstalk with different noncoding RNAs in CRC. Various ncRNA species like miRNAs, lncRNAs, and circRNAs regulate the Hippo pathway components. Specific miRNAs can inhibit YAP/TAZ, while lncRNAs act as ceRNAs to sequester these miRNAs and derepress YAP/TAZ. CircRNAs also modulate Hippo pathway activity through miRNA sponging. Dysregulation of ncRNAs that target Hippo signaling contributes to CRC progression.

This section delves into the interplay between ncRNAs and the constituents of the Hippo–YAP/TAZ signaling pathway, carrying profound implications for the biology of CRC (Figure 4).

### 5.1. miRNAs

MiR-195-5p has been associated with the prognosis of CRC, functioning as a potent suppressor of YAP1. Notably, in a murine CRC xenograft model, miR-195-5p exhibited a substantial reduction in tumor growth by targeting and downregulating YAP1. These findings imply that miR-195-5p holds potential as a valuable prognostic indicator for predicting the clinical outcomes of CRC patients [135]. By targeting TAZ, miR-125 inhibits the proliferation and invasion of CRC [136]. By downregulating YAP1, miR-375 decreased the expression of downstream genes, including CTGF, cyclin D1, and BIRC5, which led to reduced chemoresistance and better prognosis in CRC patients. Thus, miR-375 may be a valuable molecular biomarker for CRC patients and a promising therapeutic approach for those with chemoresistance [137]. The decreased expression of miR-874-3p has been implicated in the resistance of CRC cells to 5-Fluorouracil (5-FU) chemotherapy. This reduction in miR-874-3p levels contributes to the targeting of YAP/TAZ by miR-874-3p, consequently leading to the inactivation of the Hippo signaling pathway [138]. The miRNA known as miR-590-5p is responsive to changes in cell density and has the ability to directly target YAP1, thereby impeding the development of CRC tumors [139]. The direct regulation of LATS2–YAP/TAZ activation by miR-429 implies that targeting the miR-429-LATS2–YAP/TAZ axis could serve as a potential diagnostic and therapeutic approach for CRC [140].

In summary, miRNAs play a crucial role in regulating the Hippo pathway in CRC. Dysregulation of miRNA expression can lead to aberrant activation or inhibition of the Hippo pathway, ultimately contributing to the development and progression of CRC.

### 5.2. lncRNAs

B4GALT1-AS1 is a lncRNA that positively regulates colon cancer cell stemness by directly binding to YAP and enhancing YAP transcriptional activity [141]. Additionally, the lncRNA LINC00174 experiences upregulation through the transcription factor STAT1 in CRC cells. LINC00174 functions as a competitive endogenous RNA (ceRNA) for miR-1910-3p, essentially trapping it and hindering its capacity to suppress TAZ expression. This leads to the activation of the TAZ signaling pathway, facilitating the advancement of CRC. Consequently, LINC00174 emerges as a prospective therapeutic target for addressing CRC [142]. LINC00152, also known as CYTOR, is an example of a long intergenic noncoding RNA that shows high expression in CRC. YAP has the potential to target and regulate the transcription of LINC00152, which in turn affects the expression of the fascin actin-binding protein 1 (FSCN1). This promotes the development and metastasis of CRC [61]. Overexpression of YAP1 and lncRNA MALAT1 promotes the progression of CRC by inducing EMT and angiogenesis. Upregulation of YAP1 and MALAT1 is correlated with poor prognosis in CRC patients, and the silencing of these genes could inhibit tumor growth, EMT, and angiogenesis *in vivo* and *in vitro*. Targeting YAP1/MALAT1 axis may have therapeutic potential for treating CRC [143]. The modulation of the Hippo/YAP1 signaling pathway by the lncRNA USP2-AS1 is a crucial factor in the promotion of colon cancer progression. This is achieved through the interaction between USP2-AS1 and LATS1, which leads to the activation of YAP1, thereby promoting colon cancer cell proliferation, migration, and invasion [144]. Furthermore, the lncRNA GAS5 engages with YAP, prompting its phosphorylation and subsequent degradation. This process results in the suppression of CRC cell proliferation, migration, and invasion. Consequently, the modulation of the GAS5/YAP axis could present a fresh and innovative approach for CRC therapy [145].

In summary, these studies findings indicate that lncRNAs significantly impact the regulation of the Hippo signaling pathway in CRC and may serve as promising targets for developing innovative cancer treatments.

### 5.3. circRNAs

Several studies have implicated circRNAs in regulating the Hippo signaling pathway in CRC. For example, circPPP1R12A, Hsa_circ_0128846, and circ_0106714 have been shown to regulate the proliferation and migration of CRC cells through the Hippo/YAP signaling pathway [146–148]. Circstk3, also known as Hsa_circ_0004592, can regulate MST2 and promote CRC metastasis by triggering EMT as a positive regulator. These findings provide novel insights into understanding the metastatic behavior of CRC, and circSTK3 holds the potential to be a promising biomarker as well as a potential target for developing anti-metastasis treatments for CRC [149].

These findings propose a significant role for circRNAs in the regulation of the Hippo signaling pathway in CRC, potentially offering targets for novel cancer therapies. However, further research is required to comprehensively uncover the mechanisms through which circRNAs modulate the Hippo pathway in CRC.

## 6. Gut Microbiome and Immune Cells Interaction With the Hippo Pathway in CRC

Recent studies have investigated the connection between CRC and the human intestinal microbiota, consisting of many microbes ranging from 10^13^ to 10^14^, encompassing 10 times more bacterial cells than human cells and containing over 100 times as many genes as the human genome. These studies have primarily centered on investigating the potential contributions of the intestinal microbiota to the progression of CRC [150–152]. The gut microbiota plays an essential role in maintaining gut homeostasis and regulating immune function. Dysbiosis, or an imbalance in the gut microbiota, has been linked to numerous diseases, including inflammatory bowel disease, CRC, and obesity [153, 154].

Research has demonstrated that individuals with CRC exhibit a discernibly different composition of gut microbiota in comparison to healthy individuals. Notably, there is an augmentation of particular bacteria, including *Fusobacterium nucleatum*, alongside a reduction in beneficial bacteria such as *Bifidobacterium* and *Lactobacillus*, within CRC patients [155, 156].

The Hippo pathway exerts influence over the gut microbiota through various mechanisms, with one of the central processes being the modulation of intestinal stem cell (ISC) proliferation and differentiation. The Hippo pathway is activated in ISCs and regulates their proliferation and differentiation into different types of intestinal cells. Studies have shown that the Hippo pathway also regulates the differentiation of paneth cells [157], which are specialized cells in the gut that produce antimicrobial peptides that protect against pathogens [158].

In addition to regulating ISC proliferation and differentiation, the Hippo pathway regulates the expression of genes involved in host–microbe interactions. For example, the transcriptional coactivator YAP, a downstream target of the Hippo pathway, regulates the expression of antimicrobial peptides and mucins that maintain the gut barrier function and protect against pathogens [159].

The gut microbiota has therapeutic potential, with *Streptococcus thermophilus* being an example. *S. thermophilus* increases the levels of *Bifidobacterium* and *Lactobacillus* in the gut by producing galactose through *β*-galactosidase. This disrupts energy balance, stimulates oxidative phosphorylation, and suppresses Hippo pathway kinases, contributing to *S. thermophilus*' anticancer properties [160].

**Figure 5:**
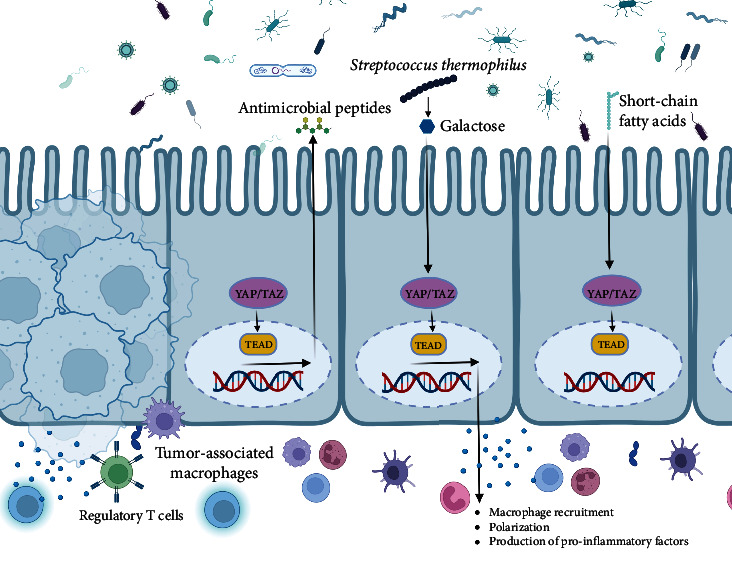
The image illustrates the interaction between gut microbiota, the Hippo signaling pathway, and the tumor immune microenvironment (TIME) in colorectal cancer (CRC). *S. thermophilus* promotes beneficial bacteria and produces antimicrobial peptides and SCFAs, activating the Hippo pathway via YAP/TAZ. Hippo signaling pathway influences macrophage recruitment, polarization, and pro-inflammatory factor production. The TME, with tumor-associated macrophages (TAMs) and regulatory T cells (Tregs), suppresses the immune response and supports tumor growth.

Conversely, recent research has highlighted that the gut microbiota possesses the ability to both activate and deactivate the Hippo signaling pathway (Figure 5), thereby exerting substantial influence over host physiology and disease outcomes. One plausible mechanism through which the gut microbiota exerts this effect involves the generation of short-chain fatty acids (SCFAs), including butyrate [161]. Butyrate has been shown to activate the Hippo signaling pathway by inhibiting the histone deacetylase (HDAC) enzyme activity, which leads to the upregulation of the Hippo signaling pathway genes [162].

To summarize, the intricate interplay between the gut microbiota and the Hippo signaling pathway constitutes a multifaceted and dynamic process that holds significant implications for the development and progression of CRC. Further research is needed to better understand the underlying mechanisms and identify potential therapeutic targets for CRC.

The tumor immune microenvironment (TIME), comprising the surroundings of tumor cells or tumor stem cells, plays a vital role in tumor onset, development, and spread. It fosters angiogenesis and attracts regulatory T cells (Tregs) and myeloid-derived suppressor cells (MDSCs) that stifle the immune system's antitumor response and boost tumor growth. Additionally, cytokines in the TME regulate immune functions and contribute to weakened immune responses, fueling tumor progression. Consequently, the TME of CRC holds immense significance in influencing the success of cancer immunotherapy [140]. Furthermore, the TME undergoes substantial influence from the presence of tumor-associated macrophages (TAMs), which play a pivotal role in tumor proliferation, metastasis, invasion, and evasion of the immune system [163]. YAP, the Hippo signaling pathway key component (Figure 5), affects macrophage recruitment, polarization, and production of pro-inflammatory factors [164].

TAZ, a component of the Hippo pathway, has been recognized as a valuable prognostic biomarker and a potential therapeutic target in CRC, owing to its association with immune infiltration [165]. Additionally, the expression of YAP1 and PTEN has been associated with the expansion of TAMs and reduced survival rates in CRC. Therefore, targeting TAMs and the Hippo pathway, particularly TAZ, may be promising strategies for improving treatment outcomes in CRC patients. Furthermore, YAP1 and PTEN expression could be useful biomarkers for predicting the progression and outcome of CRC [18].

## 7. Conclusions

To conclude, the Hippo signaling pathway has garnered significant importance in the context of CRC development and advancement. Its disruption due to various upstream signals, including cell polarity and mechanotransduction, along with the involvement of ncRNAs and the gut microbiota, underscores its potential as an attractive target for therapeutic interventions in CRC. The interplay between the Hippo pathway and various other cellular signaling pathways, such as Wnt, TGF-*β*, Notch, and the TME, offers intriguing prospects for the development of novel and innovative cancer therapies. Targeting regulators upstream of the Hippo pathway or its core kinase cascade presents opportunities to correct Hippo signaling dysfunction in CRC. Further research is necessary to gain a better understanding of the mechanisms underlying these interactions and to identify novel therapeutic targets that can improve CRC patient outcomes.

## Data Availability

Data sharing not applicable to this article as no datasets were generated or analyzed during the current study.

## Nomenclature

5-FU: 5-fluorouracil
AMOT: Angiomotin
AMOTL1: Angiomotin like-1 protein
AMPK: AMP-activated protein kinase
ceRNA: Competing endogenous RNA
circRNAs: Circular RNAs
CMS4: Consensus molecular subtype 4
CRAD: Actin dynamics
CRC: Colorectal cancer
CSCs: Cancer stem cells
Ds: Dachsous
ECM: Extracellular matrix
EGF: Epidermal growth factor
EGFR: EGF receptor
EMT: Epithelial-mesenchymal transition
Ex: Expanded
FSCN1: Fascin actin-binding protein 1
Ft: Protocadherins fat
GPCRs: G protein-coupled receptors
HDAC: Histone deacetylase
IGF-1: Insulin-like growth factor 1
ISC: Intestinal stem cell
KIRREL1: Kirre-like nephrin family adhesion molecule 1
LATS1/2: Large tumor suppressor kinase 1/2
lncRNAs: Long noncoding RNAs
MAPK: Mitogen-activated protein kinase
MDSCs: Myeloid-derived suppressor cells
Mer: Merlin
miRNAs: microRNAs
MOB1A/B: MOB kinase activator 1A/B
MSI: Microsatellite instability
MSS: Microsatellite stable
MST1/2: Sterile 20-like 1/2
ncRNAs: Noncoding RNAs
Nrf2: Nuclear factor erythroid 2-related factor 2
OS: Overall survival
PAR2: Proteinase-activated receptor 2
PCP: Planar cell polarity
PP2A: Protein phosphatase 2A
ROCK: Rho-associated protein kinase
ROS: Reactive oxygen species
SAV1: Salvador homolog 1
SCFAs: Short-chain fatty acids
snoRNAs: Small nucleolar RNAs
SOD: Superoxide dismutase
STRN: Striatin
TAMs: Tumor-associated macrophages
TGF-*β*: Transforming growth factor beta
TIME: Tumor immune microenvironment
TME: Tumor microenvironment
Tregs: Regulatory T cells
Trx: Thioredoxin
YAP/TAZ: Yes-associated protein/transcriptional co-activator with PDZ binding motif
ZO: Zonula occludens.
